# A Fast and Parallelized Procedure for Checking Molecular Dynamics 3D Structure

**DOI:** 10.1002/jcc.70494

**Published:** 2026-09-03

**Authors:** Daniel R. Roe, Bernard R. Brooks

**Affiliations:** ^1^ Laboratory of Computational Biology, National Heart Lung and Blood Institute, National Institutes of Health Bethesda Maryland USA

**Keywords:** 3D structure check, molecular dynamics, trajectory analysis

## Abstract

Structures built for or generated from Molecular Dynamics simulations can often have problems such as wrong chiralities, bad parameter assignment, steric clashes, and/or distorted geometries. It is important to be able to detect such problems in a rapid and automated way since such issues can prevent subsequent simulations from running successfully. Here we detail a structure check procedure which focuses on checking for some of the more common structural issues, including atomic overlaps, distorted bonds, and ring intersections, as well as the strategies for parallelizing the check procedure so it can rapidly detect issues in structures with millions of atoms.

## Graphical abstract

Common structural issues such as atomic overlaps, abnormal bond lengths, and ring‐bond intersections can be rapidly detected in the pairlist‐enabled and parallelized “checkstructure” command from CPPTRAJ, for example, the ~256 k atom 5a9z system can be checked in less than 2 s using 16 threads.

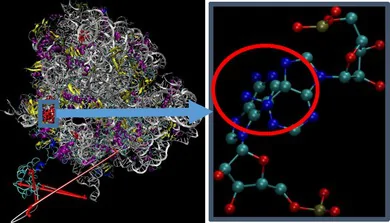

## 1 Introduction

Molecular dynamics (MD) simulations involve modeling the 3D coordinates of biological systems and other related structures. In the course of building, simulating, or otherwise manipulating these systems, it is possible for problems with the structures to be introduced. These errors are typically introduced accidentally by the user (or potentially a bug in the software being used to build the system) during construction of the system, although it is sometimes possible for preexisting issues to be present in structures determined by experimental means. While a simple visual inspection for structural issues may suffice for smaller systems, for large systems, it can be difficult or impossible to identify problems with the structure in such a manner. Similarly, if large numbers of structures need to be generated, it could take a prohibitively long time to visually inspect each structure individually. Therefore, it is useful to have a fast and automated way of identifying common structural issues in 3D coordinates.

For example, when building systems for MD it is often the case that coordinates for part (or even most) of the system are unknown; it is in fact common for PDB files to have missing atoms or entire residues due to uncertainties in the underlying experimental methods [1]. While it is sometimes prudent or acceptable to leave out residues that are missing (such as C‐terminal histidine tags in proteins), it is often necessary to build or somehow otherwise model in missing atoms. However, depending on the robustness of the build method and the quality of the structure that is present, sometimes atoms can be built in a way that either introduces a steric clash, a bond distortion, or ring‐bond skewering/intersection (when a bond between two atoms is located within another group of atoms forming a ring, essentially “locking” that bond within the ring). Atoms can also be built in such a way that they end up having the wrong chirality.

Another potential issue that can arise is periodic boundary artifacts arising from improper unit cell parameters. If the initial unit cell is too small, collisions between, for example, solvent molecules and images can occur which will cause large forces and likely unstable simulations. It is also possible to create unit cell issues if, for example, the system coordinates are rotated (via a root‐mean‐square best‐fit or other alignment) but the unit cell vectors are not similarly rotated. In addition, if solvent is not added properly, it can result in voids or “bubbles” in the system which need to be filled or collapsed in some manner.

There are various other issues that can arise that are related to parameters. For example, if the molecule has a net charge it may be the case that counterions are needed, or if the net charge of the molecule is not an integer there may be problems with the underlying charge assignment. Titratable residues that are not assigned the correct protonation state can also be problematic since protonation states can play a large roll in structural stability and dynamics [2]. Incorrect assignment of atom hybridization can lead to too many or too few bonds to a given atom (e.g., an SP3 carbon having only two or three bonds is problematic). A related issue is unexpected linearity in dihedrals (i.e., the A‐B‐C and/or B‐C‐D angle of atoms A‐B‐C‐D is linear or close to linear, resulting in an undefined torsion), which often indicates a distorted or otherwise incorrect structure. It can also be a problem if required parameters are missing; for example, if an atom has a planar hybridization but there is no dihedral or improper term to maintain planarity. Table 1 provides a summary of some of these common problems encountered when building systems for MD simulations and their source.

**TABLE 1:** Summary of some common problems encountered when building a system for molecular dynamics simulations.

Problems	Types
Steric clash	Structural
Distorted bond	Structural
Ring skewering/intersection	Structural
Incorrect atom chirality	Structural
Unexpected dihedral linearity	Structural
Small/distorted unit cell	Structural
Solvent voids	Structural
System not neutral	Structural/parameter
Net charge not integral	Parameter
Incorrect protonation state	Parameter
Incorrect atom hybridization	Parameter
Missing parameters	Parameter

Several software packages and programs include some ways of checking geometric structure. PROCHECK can be used to validate structures by checking bond lengths, angles, and torsions [3]. WHAT IF is a versatile collection of routines that can be used to both validate structure and perform homology modeling [4]. The MolProbity program detects steric clashes using an all‐atom distance‐based contact analysis, as well as evaluates deviations from optimal bond lengths and angles [5]. The NAOMI program performs several checks when converting file formats including bond order and connectivity, valence checking, and stereochemistry checks to name a few [6]. CHARMM has the “CORR DIST” command with optional “CLOSe” keyword for determining close/overlapping atoms (by identifying pairs with a positive van der Waals energy) [7]. Gromacs includes the “gmx check” command which checks for close contacts and atoms outside the box [8]. Chimera has a command which can find clashes and bad contacts [9]. VMD includes commands to check and fix chirality issues, as well as cis‐peptide bonds [10]. MDtraj has the Compute.neighbors function which allows calculation of all atoms with inter‐atomic distances smaller than a given cutoff [11]. MDanalysis has the self_distance_array command; however, it is up to the user to write more code to process the calculated distances in order to determine if atoms are “in contact” [12].

The analysis program CPPTRAJ [13] and its predecessor PTRAJ have had a “checkstructure” command for years. In its initial incarnation, the command checked for abnormal bonds (i.e., bonds longer or shorter than the equilibrium value by some distance cutoff) and overlapping atoms (i.e., two atoms closer than a certain distance). Over the years, the “checkstructure” command in CPPTRAJ has had its functionality and efficiency improved dramatically. The modern version of “checkstructure” includes the check for abnormal bonds, an improved overlap check which has been parallelized with OpenMP and made more scalable by the inclusion of a pair list (referred to hereafter without the space as “pairlist”), and a novel ring‐intersection check which looks for rings which may have been built around other bonds (which is not possible for a minimization to fix). This manuscript will describe the implementation details of the “checkstructure” algorithm, as well as some examples which illustrate how this command can be used on structures that contain millions of atoms. CPPTRAJ is open source and freely available on GitHub at https://github.com/Amber‐MD/cpptraj, and is also distributed as part of the free‐to‐download AmberTools package (https://ambermd.org).

## 2 Methods

There are three check types considered here: bonds, overlaps, and ring intersections. Each check type has a Setup phase (done once per Topology) and an Action phase (done once per coordinate trajectory Frame). In all check types, when comparing distances to a cutoff, the square of the distance is compared to the squared cutoff value to avoid calculating a square root.

Both the overlap and ring intersection checks have been sped up by making use of atomic pairlists. Atomic pairlists are a way to speed up pairwise calculations in three dimensions by dividing the space into a regular grid and only performing calculations between atoms in neighboring voxels [14, 15]. The number of voxels in a given system is determined by a cutoff within which distances of interest are to be calculated. By convention a number of voxels is chosen so that approximately 3 voxels will fit within the cutoff distance (this is similar to how the rest of Amber employs pairlists). When determining neighboring cells, cells are “wrapped,” that is, a cell at 0, 0, 0 would have the cell at −1, 0, 0 (translated to NX, 0, 0) as a neighbor. If a neighboring cell is “wrapped” in this way, the translation vector needed to wrap is stored as well, which simplifies imaging the distance calculation.

Note that although all of the examples in the current study were performed on single frames, the “checkstructure” action can be run on multiple frames (i.e., entire coordinate trajectories). Any problems detected are prefaced by the trajectory frame number where they occur in the output. The total number of problems detected per frame are saved and can be used to quickly find problematic frames in a trajectory. In addition, CPPTRAJ has a “skipbadframes” option that can be used to skip input trajectory frames where the number of detected problems is greater than 0.

### 2.1 Bond Check Setup

For each bond present in the Topology for which both atoms are selected, an equilibrium value is assigned. If bond parameters are present, this is just the bond equilibrium parameter (Req). If no bond parameters are present, a bond equilibrium value will be assigned based on the underlying atomic elements [16, 17, 18].

### 2.2 Bond Check Action

For each bond that has been set up, the non‐imaged distance is calculated between the two bond atoms. If the distance is greater than the bond equilibrium value plus the bond max offset (default 1.15 Å) or the distance is less than the bond equilibrium value minus the bond min offset (default 0.5 Å), a problem is registered that includes the identities of the bonded atoms and the distance. The defaults were empirically chosen and represent typical bond length delta values at which the difference from equilibrium starts to become significant in terms of energy. Both cutoffs can be adjusted by the user.

### 2.3 Check Overlaps Setup

There are two kinds of overlap checks: when unit cell parameters are present, a pairlist is used; otherwise, a straightforward *N* to *N* − 1 nested loop is used. In either case, for each selected atom, an atom exclusion list is set up that excludes atoms bonded to that atom (since those are checked in the Bond Check step).

### 2.4 Check Overlaps Action

#### 2.4.1 No Pairlist

When no pairlist is used, overlaps are checked using a straightforward outer (i) loop over all N atoms to inner (j) loop of all atoms with higher index, that isfor (i=0; i < N; i++)  for (j=i+1; j < N; j++)


The inner (j) loop is broken into two phases. First the atoms excluded from interacting with atom *i* are checked for, then everything else. This leverages the fact that bonded excluded atoms tend to be close in sequence.

#### 2.4.2 With Pairlist

When a pairlist is used, first the selected atoms are placed into grid voxels. The coordinates are first converted into fractional space in order to accommodate non‐orthogonal cells. Overlaps are then checked using an outer (i) loop over all M grid cells to an inner loop over all neighboring grid cells. First, overlaps are checked between all atoms in grid cell i. When checking neighboring cells, only cells in the “forward” (i.e., the +X, +Y, and +Z directions) are checked in order to avoid double‐counting interactions. The maximum number of cells to be checked in any given direction (the cell offset) was set at 3, consistent with existing pairlist implementations in Amber, CHARMM, Gromacs, and so on. All cells in the +X direction (up to the cell offset, total of three cells) are checked, all cells in the +Y direction (2*offsetX+1 = 7 cells in the 3 + Y rows, total of 21 cells), and all cells in the +Z direction (2*offsetX+1 by 2*offsetY+1 = 49 cells in the 3 +Z slabs, total of 147 cells); this means each cell has 171 neighbor cells. Cells with no atoms are skipped. Before the distance calculation is attempted, the atom exclusion list is searched to make sure this is not an excluded interaction. Due to the way neighboring cells are set up, the distances calculated correspond to the minimum imaged distance. If the distance is less than the cutoff (default 0.8 Å), a problem is registered that includes the identities of the bonded atoms and the distance. This default cutoff was chosen to be consistent with results from PTRAJ [13] and is user adjustable.

### 2.5 Check Ring Intersections Setup

For all selected atoms, groups of atoms that form rings are searched for within residues (i.e., it is assumed that rings do not span residues). First, a list of atoms which can potentially start a ring is made; these are atoms with more than one bond. For each potential start atom, that atom (at1) and an atom bonded to it in the same residue (at2) are chosen. A recursive search is then performed to see if there is a path within this residue that connects the two atoms. If a ring is found, the ring atoms are recorded. This is repeated until there are no more potential start atoms to check. Only the shortest possible rings are used; for example, in a Tryptophan residue two rings will be detected, one with six atoms and one with five atoms.

Once rings are found for a residue with a given name, the information is cached so that the ring search is only done once for a given residue name. This was found to be necessary for structures with high residue counts (100 k residues or more) since the ring search is relatively computationally expensive. Once the residue name is in the ring cache, subsequent residues with the same name are checked to ensure all atoms in the cached rings exist in that residue.

The same list of bonds that is used in the Bond Check is used for the Ring Intersections Check. Like the Check Overlaps procedure, the ring check makes use of a pairlist if unit cell parameters are present. If a pairlist is to be used, it is set up in the same manner as for the Check Overlaps procedure.

### 2.6 Check Ring Intersections Action

The first step of the calculation is for each ring, calculate a vector that is perpendicular to the plane of each ring with an origin at the geometric center of the ring atoms. This is done by first calculating the least‐squares best‐fit plane through the ring atoms relative to their center of geometry following the procedure outlined by Glusker et al. [19]. Briefly, first the center of geometry of the ring atoms is computed. The coordinates of the ring atoms are then translated so that the center of geometry is at the coordinate origin. The covariance matrix *M* of these coordinates is then found.

$$M=\left\lceil \begin{array}{ccc} \sum x^{2} & \sum \mathrm{xy} & \sum \mathrm{xz}\\ \sum \mathrm{xy} & \sum y^{2} & \sum \mathrm{yz}\\ \sum \mathrm{xz} & \sum \mathrm{yz} & \sum z^{2} \end{array}\right\rceil$$

The smallest eigenvalue of *M* is then found by solving for the smallest root (*λ*_min_) of a cubic equation using Cardano's formula [20].

$$\det\left(M-\mathrm{\lambda I}\right)=0$$

$$-\lambda^{3}+o\lambda^{2}+\mathrm{p\lambda}+q=0$$

$$o=\sum x^{2}+\sum y^{2}+\sum z^{2}$$

$$\begin{array}{c} p={\left(\sum \mathrm{xy}\right)}^{2}+{\left(\sum \mathrm{xz}\right)}^{2}+{\left(\sum \mathrm{yz}\right)}^{2}\\ \;-\left(\sum x^{2}*\sum y^{2}+\sum x^{2}*\sum z^{2}+\sum y^{2}*\sum z^{2}\right) \end{array}$$

$$\begin{array}{c} q=\left(\sum x^{2}*\sum y^{2}*\sum z^{2}\right)+\left(2*\sum \mathrm{xy}*\sum \mathrm{xz}*\sum \mathrm{yz}\right)\\ \;-\left(\sum x^{2}*{\left(\sum \mathrm{yz}\right)}^{2}+\sum y^{2}*{\left(\sum \mathrm{xz}\right)}^{2}+\sum z^{2}*{\left(\sum \mathrm{xy}\right)}^{2}\right) \end{array}$$

The vector perpendicular to this best‐fit plane is then given by

$$\mathrm{VX}=\left(\sum y^{2}-\lambda_{\min}\right)*\sum \mathrm{xz}-\left(\sum \mathrm{xy}-\sum \mathrm{yz}\right)$$

$$\mathrm{VY}=\left(\sum x^{2}-\lambda_{\min}\right)*\sum \mathrm{yz}-\left(\sum \mathrm{xy}-\sum \mathrm{xz}\right)$$

$$\mathrm{VZ}={\left(\sum \mathrm{xy}\right)}^{2}-\left(\sum y^{2}-\lambda_{\min}\right)*\left(\sum x^{2}-\lambda_{\min}\right)$$

The vector is then normalized. Note that this is the same procedure used by the CPPTRAJ ***vector corrplane*** Action.

#### 2.6.1 No Pairlist

**FIGURE 1:**
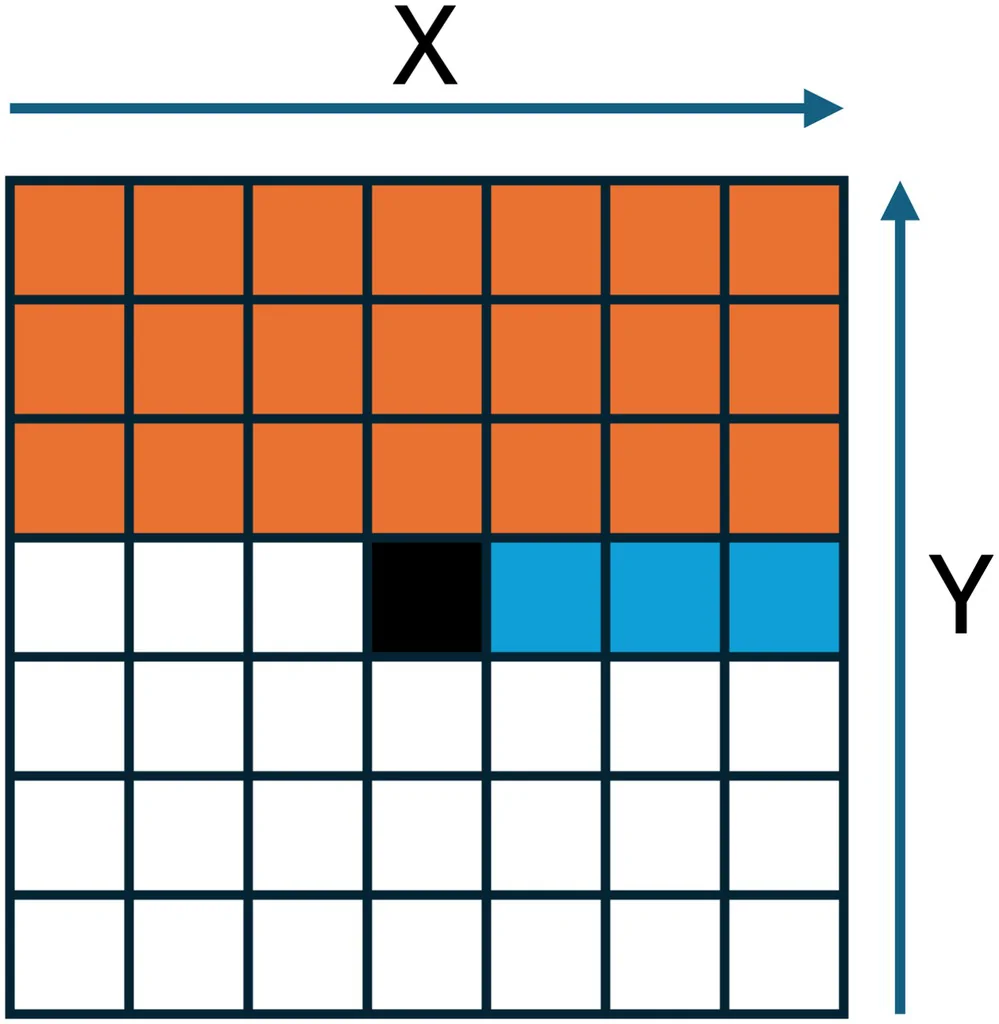
Illustration of the pairlist neighboring cell search from a center cell (black shaded cell) in the forwards direction for the *X* (blue shaded cells) and *Y* (orange shaded cells) axes. The 7 × 7 slabs along the +*Z* axis (not shown) are also searched.

If no pairlist is to be used, then for each bond the midpoint of that bond and the bond vector are calculated. Then the bond is checked against each ring that has been set up. First, the bond is checked to make sure it is not part of the ring in question. Then, the distance between the bond midpoint and ring perpendicular vector origin (i.e., the geometric center of the ring atoms) is calculated. If it is less than a distance cutoff (default 1.25 Å), the ring perpendicular vector is compared to the bond vector. If the distance is less than a stricter cutoff (default 0.5 Å), the bond is considered to be intersecting the ring. Otherwise, the angle between the normalized bond and ring perpendicular vectors is calculated (via a dot product) and wrapped between 0° and 90°. If the angle is less than a cutoff (default 65°) then the bond is considered to be intersecting the ring. This concept is illustrated in Figure 1.

If the bond is considered to be intersecting the ring, a problem is registered that includes the identities of one of the bond atoms, one of the ring atoms, and the bond midpoint to ring perpendicular vector origin distance. Note that the current defaults for ring intersection detection were chosen and adjusted based on empirical observations of several ring intersections. It is possible they require more optimization. All of the cutoffs can be adjusted by the user.

#### 2.6.2 With Pairlist

When using a pairlist for the ring intersection check, the procedure is similar to the check overlaps procedure except instead of atomic coordinates, the coordinates of the bond midpoints and ring perpendicular vector origins are placed on the same grid. The bond midpoints are added first, then the max index is recorded and the ring vector origins are added second. This way it is simple to ensure that only bond midpoint to ring vector distances are calculated (and not, e.g., two bond midpoints). Doing the distance calculation with the pairlist gives the same advantages as with the overlaps check (speed and imaging). Any bond midpoint to ring perpendicular vector that satisfies the cutoff then proceeds to compare the bond vector to ring perpendicular vector in the same manner described in the “No Pairlist” section above.

### 2.7 Considerations When Running Multi‐Threaded

When working in a multithreaded shared‐memory (i.e., OpenMP [21]) environment, some care must be taken when recording problems. In each check, the outer loop is parallelized and divided among threads. For the bond check this is the loop over the list of bonds. For the overlap check this is the outer loop over selected atoms, or the outer loop over grid cells when using a pairlist. For the ring check, the loop over bonds is parallelized when not using a pairlist, but when using a pairlist there are two phases. First, the loop to calculate the bond vectors and midpoints is parallelized, then the outer loop over grid cells is parallelized. For each check, the problem data structure has a common format: two integers and one double precision float. In order to avoid clashing in memory, each thread writes problems to a separate array. When the calculation is over for a given input coordinate frame, the problems from individual threads are consolidated to a main problem array one thread at a time, and then to ensure consistency between different thread counts the consolidated list of problems is sorted (first by Problem Atom 1, then by Problem Atom 2).

### 2.8 Test Systems

The “checkstructure” calculation was tested on a variety of systems. All systems were built using Cpptraj and AmberTools [22]. A summary of the test system sizes is given in Table 2.

**TABLE 2:** Test systems used, along with the experimental method used and resolution. 1LE1 was obtained with high‐precision NMR and does not have a resolution value.

PDB ID	# PDB Atoms	Resolution (Å)	Method
1LE1	220	n/a	NMR
3nrc	4096	2.1	X‐Ray
7a5a	23,452	2.99	X‐Ray
4kvb	51,128	4.2	X‐Ray
1jj2	98,543	2.4	X‐Ray
5a9z	150,547	4.7	Elec. Microscopy
7ane	303,111	3.9	Elec. Microscopy
6o3h^a^	1,871,280	2.8	Elec. Microscopy

^a^ Original PDB has 60 individual MODELs (31,188 atoms per MODEL) which are used to assemble the final system.

## 3 Results and Discussion

### 3.1 Choosing the Pairlist Cutoff

**FIGURE 2:**
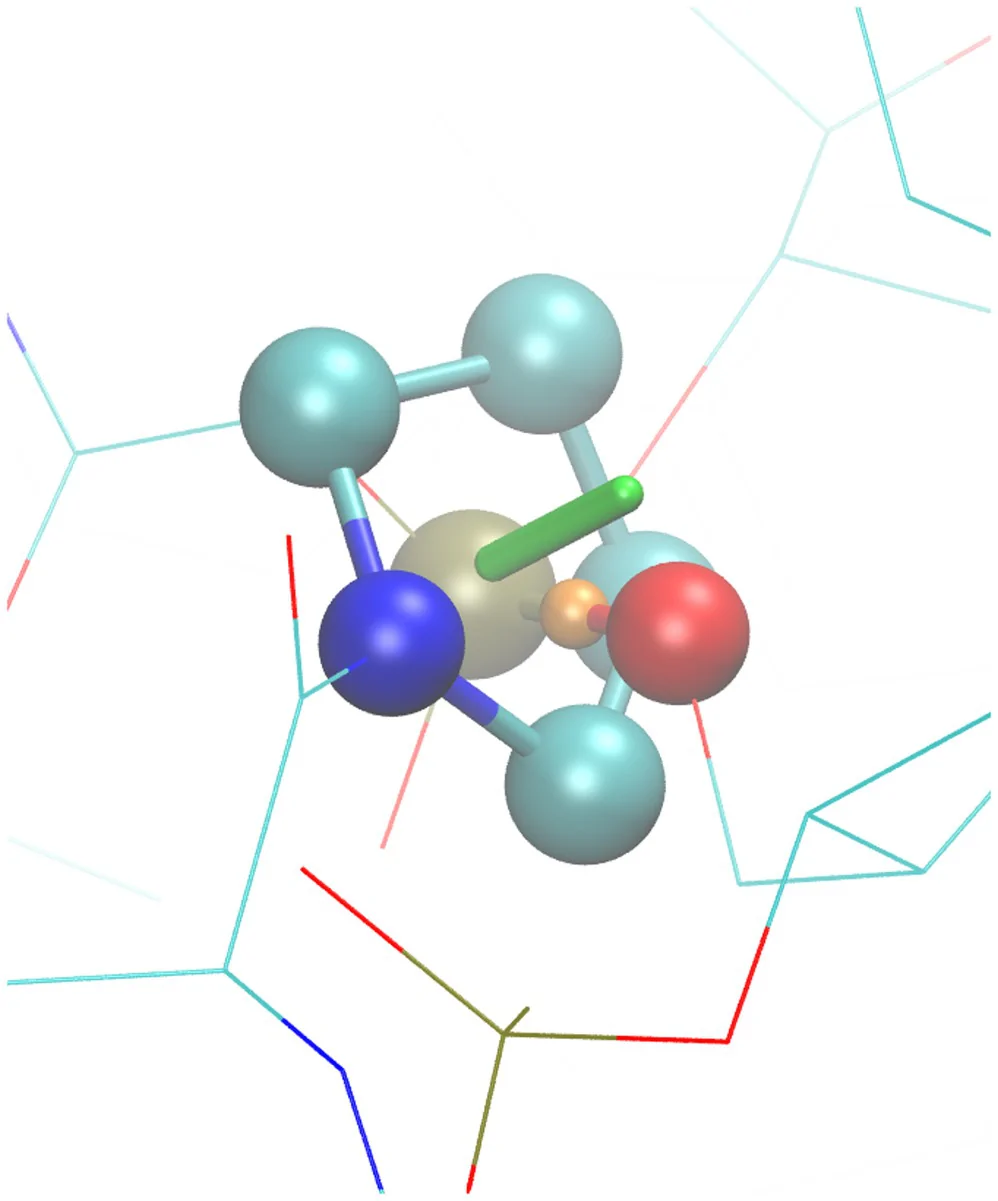
Illustration of the ring intersection calculation for a structure using PDB 7ANE. The ring atoms of proline 17,585 (N, CA, CB, CG, CD) and bond atoms of uracil 13 (P, O5′) are shown in CPK representation. The midpoint of the bond is shown as an orange sphere. The vector perpendicular to the plane of the ring is shown as a green cylinder.

When choosing a cutoff size, there is a balance to be struck between calculation speed and memory requirements: a shorter cutoff is typically faster since there are fewer elements per voxel on average, but the overall memory requirements are larger since more voxels can fit within the unit cell. Conversely, a larger cutoff tends to be slower but uses less memory. The default pairlist cutoff of 8.0 Angstroms in CPPTRAJ was chosen as a good compromise between speed and memory usage for typical solvated system sizes (up to approximately 262,000 Å^3^). Figure 2 illustrates the tradeoff between calculation speed and memory usage for various pairlist cutoff distances. Even though the minimum distance being searched for is quite small (typically < 2 Å), a cutoff of 8 Å dramatically reduces the memory needed by the pairlist grid while maintaining a tractable calculation time.

**FIGURE 3:**
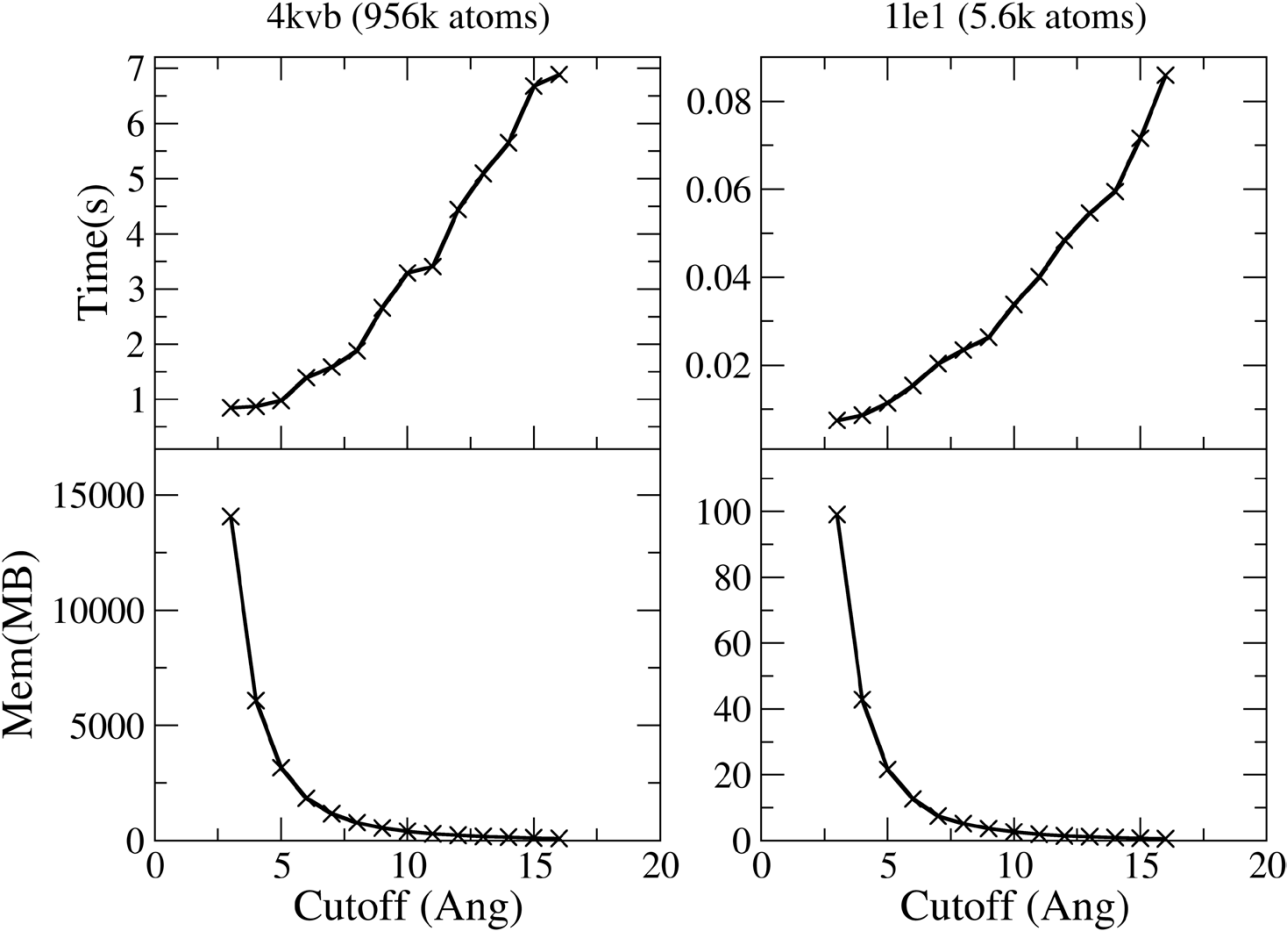
Check overlaps calculation time (top) and pairlist grid memory usage (bottom) for various pairlist cutoff distances for a relatively large solvated system (PDB 4 kvb, 10,805,893 Å^3^) and a much smaller solvated system (PDB 1le1, 78,584 Å^3^). There is a dramatic decrease in memory usage as the cutoff is increased from 2 to 8 Å with a relatively small corresponding increase in calculation time. Plot generated with xmgrace 5.1.25.

However, it was found that for large systems (> ~262,000 Å^3^) the default cutoff of 8 Å still resulted in large memory usage. For example, with a cutoff of 8 Å, the 4kvb system shown in Figure 3 requires 778 MB of memory (calculation time 1.8 s). Therefore, we came up with a simple heuristic that for systems where the minimum box dimension is greater than 64 Å, the pairlist cutoff is set to be the square root of that minimum box dimension. For the 4kvb system, the minimum box dimension is ~199 Å, resulting in an automatically determined pairlist cutoff of 14.1 Å, which requires only 145 MB of memory (calculation time 5.9 s). Note that this is just the default behavior; the user is always free to choose whatever cutoff they desire.

### 3.2 Pairlist Versus Non‐Pairlist Caclulations

**FIGURE 4:**
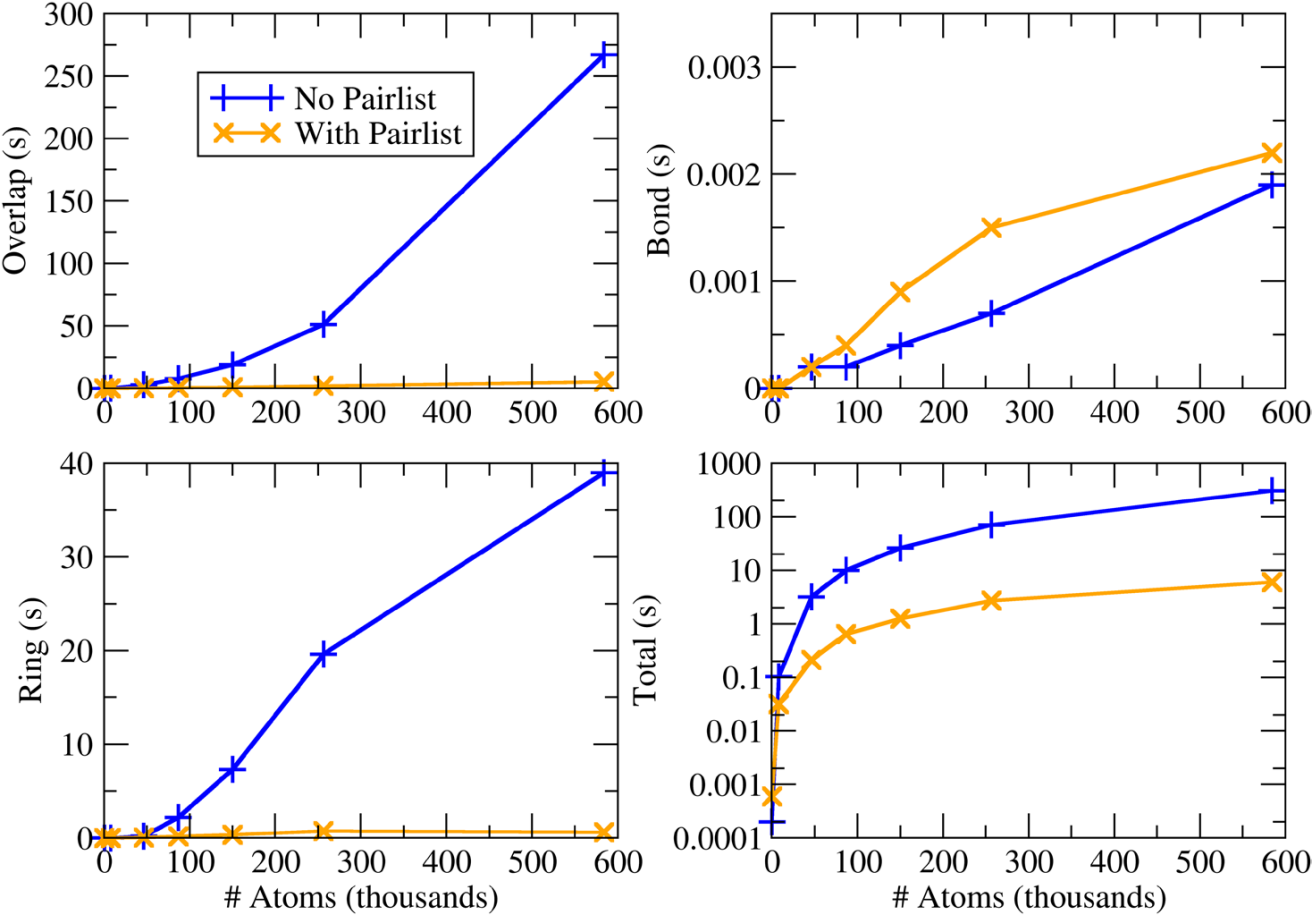
Timing results for single‐threaded (Intel Xeon Gold 6538Y+) “checkstructure” calculation in CPPTRAJ with (orange lines) and without (blue lines) the pairlist enabled. The total calculation time is broken down into the time taken for the check overlaps, bonds, and ring intersections phases. The bond calculation does not ever use a pairlist. The Total time graph has a logarithmic *Y* axis scale for improved clarity.

The pairlist‐enabled “checkstructure” calculation was compared against the non‐pairlist calculation for a variety of system sizes ranging from a few hundred to a few hundred thousand atoms. Timing results are shown in Figure 4.

The bond calculation is extremely fast for all systems tested, never taking more than 0.0022 s of calculation time, so it does not require and would not benefit from using a pairlist. The timing differences between the with and without pairlist timings for the bond calculation are the result of normal timing variance (max delta for any system is only 0.0008 s). The majority of the calculation time is taken up by the overlap and ring intersection checks, with the overlap check taking the most time. The overlap and ring intersection checks scale very poorly for increasing numbers of atoms when a pairlist is not used. For the largest system tested without the pairlist enabled (7ane, ~585 k atoms) the calculation takes about 5 min, with the overlap portion taking ~87% of the total time. In contrast, when using a pairlist that same calculation takes only 6 s, with the overlap portion again taking ~89% of the total time. This illustrates the utility and necessity of using a pairlist for the “checkstructure” calculation for large systems. Calculations for larger systems were not conducted without a pairlist since the time needed became prohibitively large.

### 3.3 Detection of Problems

The detected problems with each structure before and after building are reported in Table 3. In general, the larger the incoming system, the more problems there are both before and after building. For the small to moderately sized systems (< 200 k atoms), the main issues are that (1) when missing atoms are built in they can overlap with existing atoms (this is particularly true for hydrogens) and (2) if chains are missing one or more residues and are not truncated, an abnormally long bond can be created across the gap created by the missing residue(s).

**TABLE 3:** Number of overlaps, distorted bonds, and ring‐bond intersections detected for each test system before and after building with AmberTools. Atoms is the number of atoms in the final built system. The solvation process used in AmberTools specifically prevents atomic overlaps between solute and added solvent, so no new structure problems are introduced in the solvated systems.

#Names	Built atoms	OverlapPDB	OverlapBuild	BondPDB	BondBuild	RingPDB	RingBuild
1le1	220	0	0	0	0	0	0
3nrc	7882	0	0	0	0	0	0
7a5a^c^	46,274	0	1	0	4	0	0
4kvb	86,692	0	0	0	1	0	0
1jj2	150,010	0	5	0	14	0	0
5a9z^b^	256,302	84	584	14	17	31	32
7ane^b^	584,051	37	220	0	43	12	14
1jj2 (solvated)	1,401,458	0	5	0	14	0	0
6o3h^a^	3,709,020	0	1020	0	0	0	0
7ane^b^ (solvated)	4,922,491	37	220	0	43	12	14
6o3h^a^ (solvated)	60,655,464	0	1020	0	0	0	0

^a^ Original PDB has 60 individual MODELs which are used to assemble the final system.

^b^ Original structure too large to fit the legacy PDB format; structure is from the PDBx/mmCIF‐formatted file.

^c^ Original structure has too complex a sheet topology for legacy PDB format; structure is from the PDBx/mmCIF‐formatted file.

**FIGURE 5:**
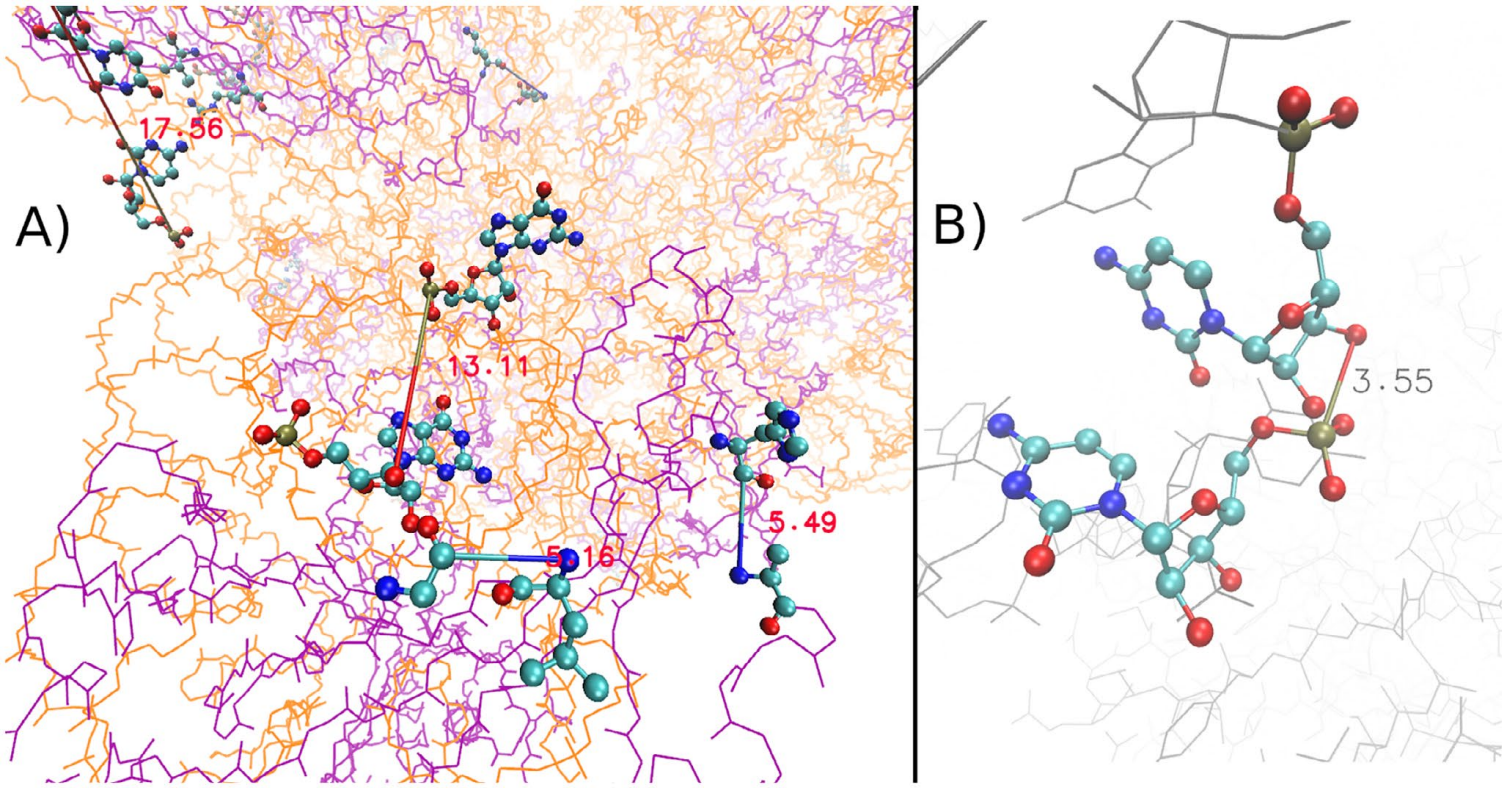
(A) Highlighting residues (in CPK representation) between which long bonds caused by gaps in the original sequence have been created in the built 1jj2 system. Protein backbone is shown in purple, nucleic acid backbone is shown in orange, abnormal bond distances (in Å) shown in red text. (B) Abnormally long bond in 5a9z between atom O3′ of residue C 884 (chain A) and atom P of residue C 885. Abnormal bond distance (in Å) show in black text.

For example, in the 7a5a system, a single overlap is introduced on building (a hydrogen atom is modeled in too close to a carbon) and four long bonds are introduced in four chains where there is a gap of six missing residues. These long bonds indicate to the user that they must either truncate the chains or model in the missing residues. In the 4kvb system, a single long bond is introduced where there is a gap of four missing residues. In the 1jj2 system there are five close contacts ranging from 0.6 to 0.8 Å from missing hydrogens being built near each other, and 14 long bonds introduced from residue gaps ranging from 1 to 100 residues in length. Figure 5A shows some of these long bonds in the final built 1jj2 structure.

**FIGURE 6:**
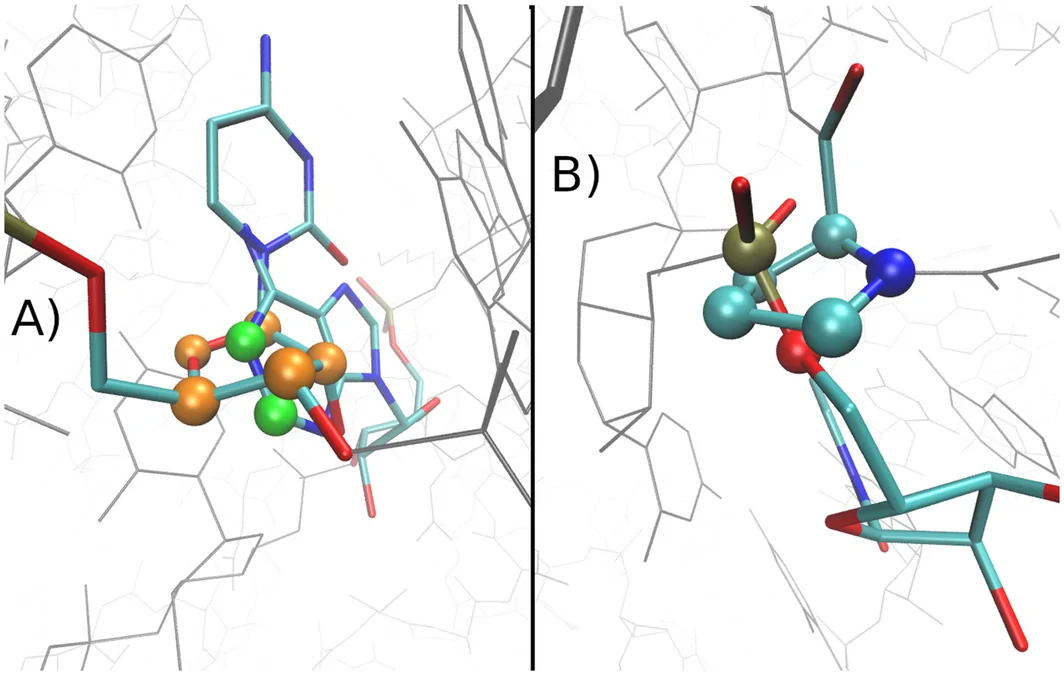
(A) Ring‐bond intersection in 5a9z involving the N1–C2 bond of residue A 8 (Chain A) and the ribose ring of residue C 2896 (Chain A). This results in the purine ring of the adenine becoming interlocked with the ribose of the cytosine. (B) Ring‐bond intersection in 7ane involving the P‐O5′ bond of residue U 13 (Chain A) and the pyrrolidine ring of residue P 262 (chain XA). Atoms involved in the problem are shown in CPK representation, atoms in the associated residues are shown in licorice representation.

In the larger (> 200 k atoms) systems, there are more problems with the initial structures. The initial 5a9z structure contains 84 overlaps, 14 abnormal bonds, and 31 ring intersections. The overlaps and abnormal bonds are the result of poor coordinate placement of existing atoms; an example of an abnormally long bond created between two cytosine residues is shown in Figure 5B. The 5a9z structure is from Cryo‐EM data with relatively low resolution (4.7 Å) so it is perhaps not surprising that there are some structure artifacts [23]. However, while it is usually possible to resolve close contacts with careful minimization, ring‐bond intersections cannot be minimized out and require direct intervention to resolve; an example of this is shown in Figure 6A. Tools like LongBondEliminator can be used to resolve ring‐bond intersections when they are detected [24].

Similarly, the initial 7ane structure contains 37 overlaps and 12 ring‐bond intersections; Figure 6B shows an example of one of the ring‐bond intersections. This structure is also from lower‐resolution (3.9 Å) Cryo‐EM data [25]. For comparison, the 4kvb system has a similarly low‐resolution (4.2 Å) X‐ray data but has no issues prior to building. It is currently unclear why these issues appear in the structures modeled from the low‐resolution Cryo‐EM data; this will be explored further in future work.

### 3.4 Calculation Speed and Parallelization

The bond, overlap, and ring‐bond checks are all parallelized with OpenMP. The bond check is parallelized by dividing the bond list among available threads. The non‐pairlist overlap check is parallelized by dividing the atoms in the outer loop. The pairlist‐overlap check is parallelized by dividing the grid voxels. There are several places where the ring‐bond intersection check is parallelized. First, the calculation of the ring vectors is parallelized by dividing up the number among available threads. The non‐PL ring‐bond intersection check is then parallelized by dividing up the number of bonds, calculating the bond midpoint, then calculating to each ring vector. The pairlist ring‐bond intersection check is parallelized by first dividing up the calculation of all the bond midpoints, then placing the bond midpoints and ring centers on the PL grid and dividing the grid voxels among available threads.

**FIGURE 7:**
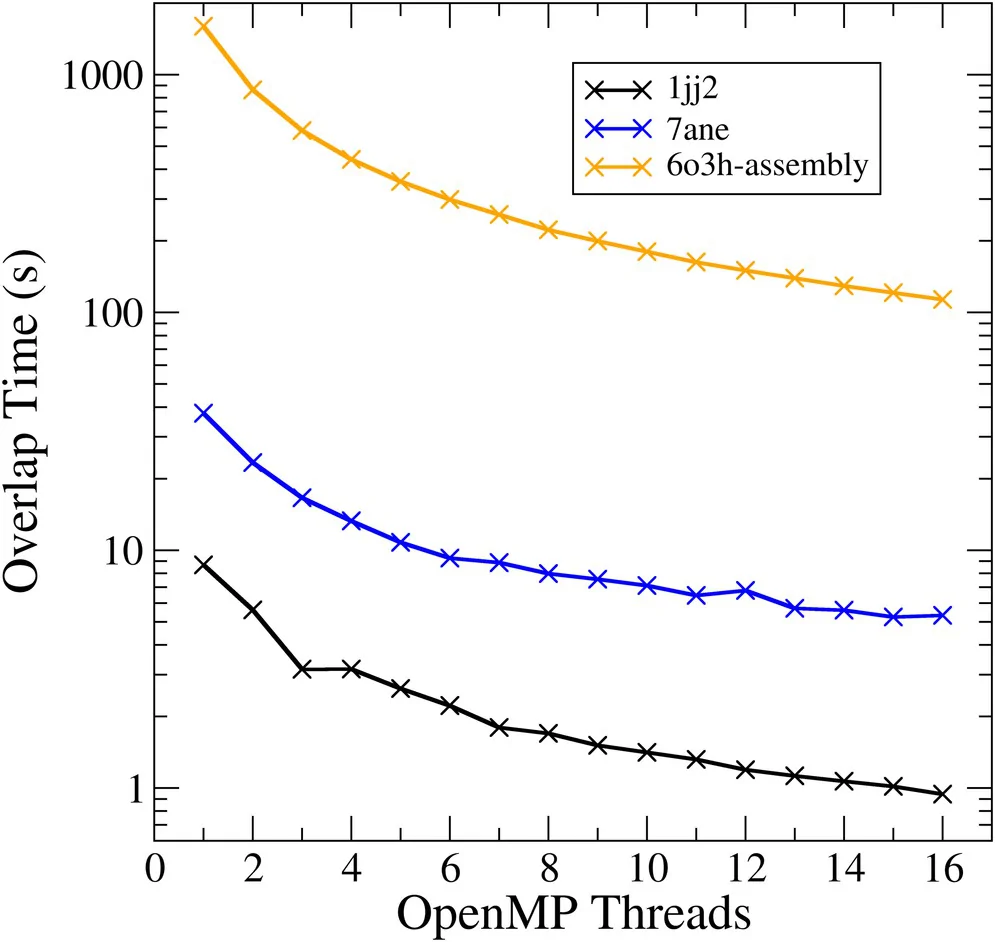
Check overlap time in seconds (log scale) versus number of OpenMP threads for three large (> 1 M atom) systems. One OpenMP thread just means serial execution. In each case, the overlap check time is the majority percentage of the total calculation time: 1jj2 = 93.2 ± 1.7%, 7ane = 96.5 ± 0.8%, 6o3h = 99.4 ± 0.2%.

A plot showing overlap check timing for three large systems, 1jj2 solvated with explicit water (1,401,458 atoms), 7ane solvated with explicit water (4,922,491 atoms), and a solvated icosahedral reconstruction of a viral capsid (assembly from PDB 6o3h, 60,655,464 atoms) is shown in Figure 7. For each system, the overlap check takes the majority (> 93%) of the total check calculation time. For the 1jj2 system with about 1.4 M atoms, the calculation is still on the order of seconds for a single thread, but for the extremely large 6o3h assembly (60 M atoms) the overlap check takes almost half an hour, emphasizing the need for parallelization. With 16 OpenMP threads the calculation takes a more manageable 114 s. It is likely that for routine calculations on trajectories of this size more speedup will be needed; future work will include adapting the check procedure code to GPUs.

**FIGURE 8:**
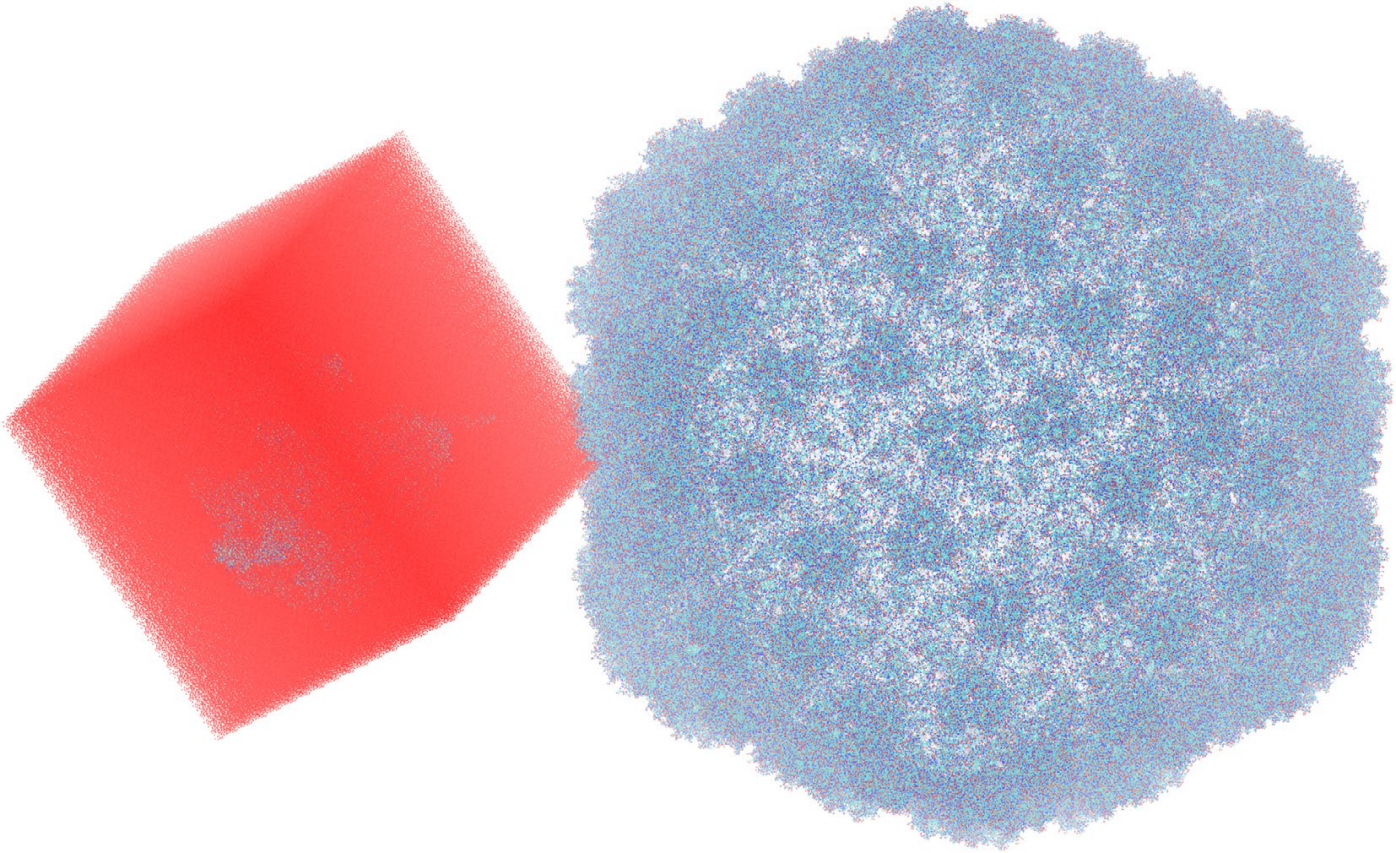
Comparison of the solvated 7ane system (left, ~4.9 M atoms) to the dry 6o3h system (right, ~3.7 M atoms). The solvated 7ane system has more atoms but is also denser, requiring fewer grid cells for the pairlist compared to the larger and less dense 6o3h system.

Another observation of note is that with a pairlist, memory usage and system size are not necessarily correlated. Table 4 shows total timings and pairlist memory usage for all systems using OpenMP with 16 threads. Although in general systems with more atoms take longer to complete and require more memory, there are some cases where systems with more atoms process faster and use less memory, such as the solvated 7ane system versus dry 6o3h; solvated 7ane is about 2 s faster and uses ~540 MB less memory despite having 1 M more atoms than dry 6o3h. The reason is that as illustrated in Figure 8 the volume of dry 6o3h is much bigger than solvated 7ane (6o3h is a viral capsid assembly with lots of empty space inside the capsid) and so more grid cells are needed for the pairlist. Although these cells end up empty, they still need to be allocated and checked. When solvent is added to the 6o3h system, the memory usage is approximately the same but the timing increases since there are many more atoms to check. It is potentially possible to increase the speed and decrease the memory requirements of checking sparse systems like dry 6o3h by switching from a 3D grid to an Octree representation. This may be explored in future work.

**TABLE 4:** Timings and pairlist memory usage for serial and 16‐threaded OpenMP pairlist‐enabled “checkstructure” runs. To obtain system volume and ensure the pairlist code was active for non‐solvated systems, a van der Waals bounding box with a 2.0 Å offset was added using the CPPTRAJ “box” action.

Systems	#Atoms	Serial (s)	OMP16 (s)	Cutoff (Å)	Pairlist Mem. (MB)	Volume (nm^3^)
1le1	220	0.0007	0.0007	8.0	0.71	12.6
3nrc	7882	0.0234	0.0082	8.0	22.30	326.4
7a5a	46,274	0.1690	0.0370	9.4	138.99	3135.1
4kvb	86,692	0.5190	0.1318	13.4	123.84	8180.0
1jj2	150,010	0.9833	0.1898	13.5	144.80	9574.4
5a9z	256,302	2.1891	0.4289	15.9	180.49	19,524.4
7ane	584,051	5.9077	1.0411	17.2	261.42	35,813.4
1jj2 (solvated)	1,401,458	9.1480	0.8287	14.2	161.28	12,376.2
6o3h	3,709,020	81.7621	6.6440	28.0	827.95	484,034.8
7ane (solvated)	4,922,491	55.1644	4.6553	17.8	285.56	42,477.4
6o3h (solvated)	60,655,464	2260.6015	172.2742	28.1	858.24	493,291.1

## 4 Conclusions

As the size of the systems that can be modeled with molecular dynamics continues to grow, it is increasingly important to have automated ways to check for structural issues that may prevent simulations from running successfully. This is particularly important in the case of ring‐bond intersections, which cannot be resolved through careful minimization and relaxation, and may be difficult to spot in large (> 100 k atom) structures. We have developed a tool to quickly check for common structural artifacts (abnormal bond lengths, atomic overlaps, and ring‐bond intersections) in an automated way. A pairlist is essential for speeding up the overlap and ring‐bond intersection checks for large systems. The calculations can be sped up even further by dividing the work among multiple threads with OpenMP.

The speed‐up obtained can be quite significant. For example, for the fully built 5a9z system (256,302 atoms), the CPPTRAJ “checkstructure” action using a pairlist was able to complete in 2.1 s on a single thread. In contrast, the MolProbity web server (http://molprobity.biochem.duke.edu) with “Clashes” and “Geometry evaluation” selected was unable to finish processing the raw PDB (150,547 atoms) even after several minutes of run time. We note that since MolProbity is running as a webserver, it is difficult to get a true “apples to apples” comparison as the underlying hardware is almost certainly different and the exact checks MolProbity is performing are not the same as CPPTRAJ. However, for the purposes of ascertaining simple structure clashes, CPPTRAJ is significantly faster.

Application of this structure check to several systems downloaded from the PDB showed that although structure artifacts can be introduced when building in missing atoms, they can also be present in the initial structures from experimental data, particularly for larger systems at low resolutions where it may be difficult or impossible to detect problems visually. This tool should help facilitate detection of such issues so that they can be resolved prior to running MD simulations. Future work will focus on more comprehensive testing of issues involving both additional structural issues (e.g., unexpected dihedral linearity) and bad parameterization (wrong hybridization/chirality etc.).

## Funding

This work was supported by the National Institutes of Health (Z01 HL001051‐27).

## Data Availability

The data that support the findings of this study are available from the corresponding author upon reasonable request.
